# Prediction of individual response to anticancer therapy: historical and future perspectives

**DOI:** 10.1007/s00018-014-1772-3

**Published:** 2014-11-12

**Authors:** Florian T. Unger, Irene Witte, Kerstin A. David

**Affiliations:** 1 Indivumed GmbH, Falkenried 88 Bldg. D, 20251 Hamburg, Germany; 2 Institute for Biology and Enviromental Sciences, Faculty V, University of Oldenburg, Oldenburg, Germany

**Keywords:** Cell culture models, Chemosensitivity assays, Genomics, Proteomics, Personalized medicine, Response to therapy

## Abstract

Since the introduction of chemotherapy for cancer treatment in the early 20th century considerable efforts have been made to maximize drug efficiency and at the same time minimize side effects. As there is a great interpatient variability in response to chemotherapy, the development of predictive biomarkers is an ambitious aim for the rapidly growing research area of personalized molecular medicine. The individual prediction of response will improve treatment and thus increase survival and life quality of patients. In the past, cell cultures were used as in vitro models to predict in vivo response to chemotherapy. Several in vitro chemosensitivity assays served as tools to measure miscellaneous endpoints such as DNA damage, apoptosis and cytotoxicity or growth inhibition. Twenty years ago, the development of high-throughput technologies, e.g. cDNA microarrays enabled a more detailed analysis of drug responses. Thousands of genes were screened and expression levels were correlated to drug responses. In addition, mutation analysis became more and more important for the prediction of therapeutic success. Today, as research enters the area of -omics technologies, identification of signaling pathways is a tool to understand molecular mechanism underlying drug resistance. Combining new tissue models, e.g. 3D organoid cultures with modern technologies for biomarker discovery will offer new opportunities to identify new drug targets and in parallel predict individual responses to anticancer therapy. In this review, we present different currently used chemosensitivity assays including 2D and 3D cell culture models and several –omics approaches for the discovery of predictive biomarkers. Furthermore, we discuss the potential of these assays and biomarkers to predict the clinical outcome of individual patients and future perspectives.

## Introduction

**Fig. 1:**
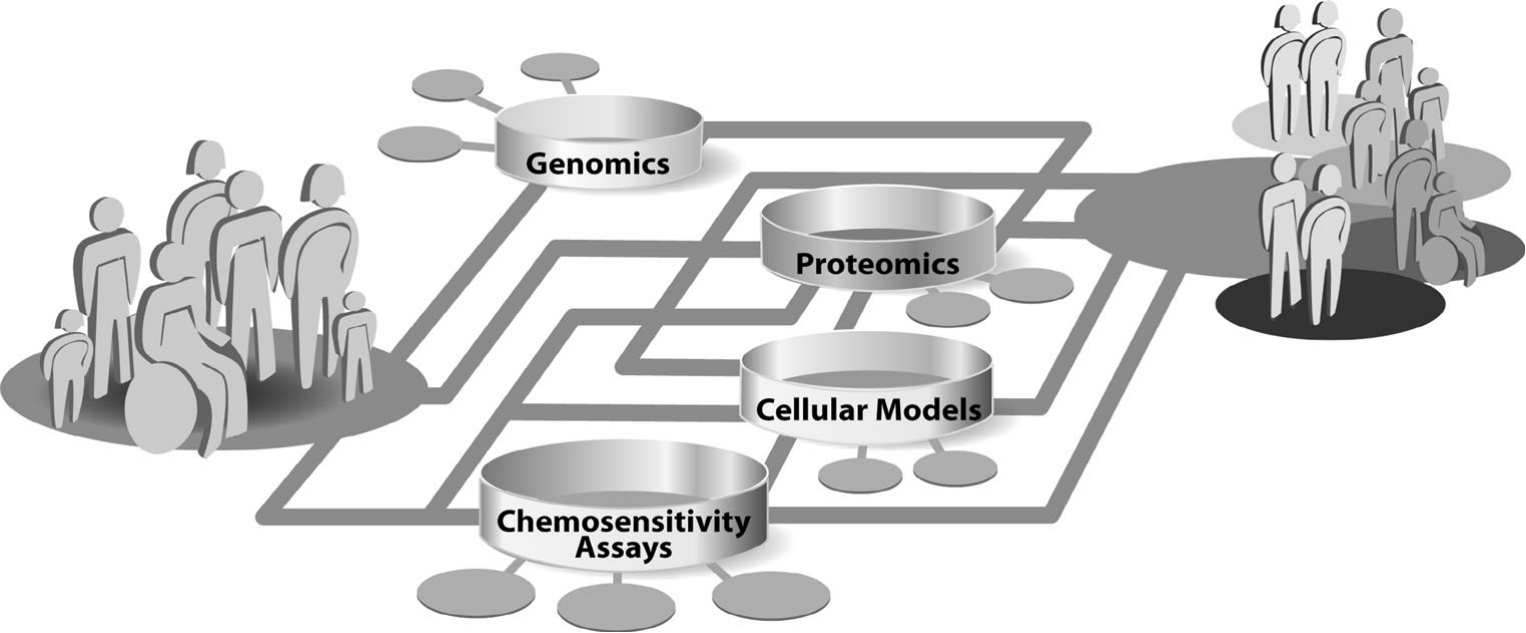
Schematic illustration of the concept of the realization of personalized medicine by molecular analysis

In the past decades, research in the field of molecular profiling of cancer was strongly affected by the rapid development of technologies. The complex disease-related alterations in the molecular networks, that are associated with response to chemotherapy, result in significant clinical heterogeneity among individual tumors and patients. A detailed and comprehensive understanding of drug response mechanisms is essential to ultimately guide a molecular based personalized anticancer therapy. Today, the complex networks of cellular mechanisms in cancer cells are just incipiently understood. Progress in all fields of cancer research, ranging from the optimization of cellular models and chemosensitivity assays over proteomics to genomics is revealing more and more facets of determinants of individual chemosensitivity. Besides studies in patients and xenograft models of tumors, in vitro cell cultures are the most commonly used systems for the analysis of cellular responses to drug treatment. A whole spectrum of cellular models ranging from secondary cell lines and primary mixed cultures over multicellular spheroids to organoid cultures are being used in cancer research. These models are being constantly optimized to mimic the origin tumor and tumor microenvironment as close as possible. Cell culture models are the basis for the molecular analysis of individual drug response. Relatively common approach to measure cellular chemosensitivity is the use of various in vitro chemosensitivity assays, which basically only detect the sum of all specific cellular drug effects. This measurement of drug effects on cell viability is deeply integrated in basic research, as well as in the clinical setting for the general determination of chemoresistance of a patients` tumor. To investigate the molecular details of individual drug responses, genomic and proteomic methods were integrated in cancer research. These technologies enable comprehensive investigation of the multi-factorial mechanisms underlying individual drug response by the simultaneous analysis of thousands of genes or proteins. This huge amount of generated data can be merged to a complex picture of molecular networks and will significantly contribute to the understanding of the diversity in individual drug response. The technical advances in all areas are enhancing the amount of information output rapidly and ultimately the interconnection of all fields of research should be able to combine molecular attributes to individual, molecular signatures of chemosensitivity. The molecular characterization of patients will shift the concept of anticancer therapy from standardized treatment of patients to specialized treatment concepts for molecular-defined subgroups of patients (Fig. 1). In the future, this individualization of anticancer therapy will increase survival and life quality of patients, by being able to provide maximal effective therapies and sparing them from uneffective therapies and side effects.

## Cellular models

**Fig. 2:**
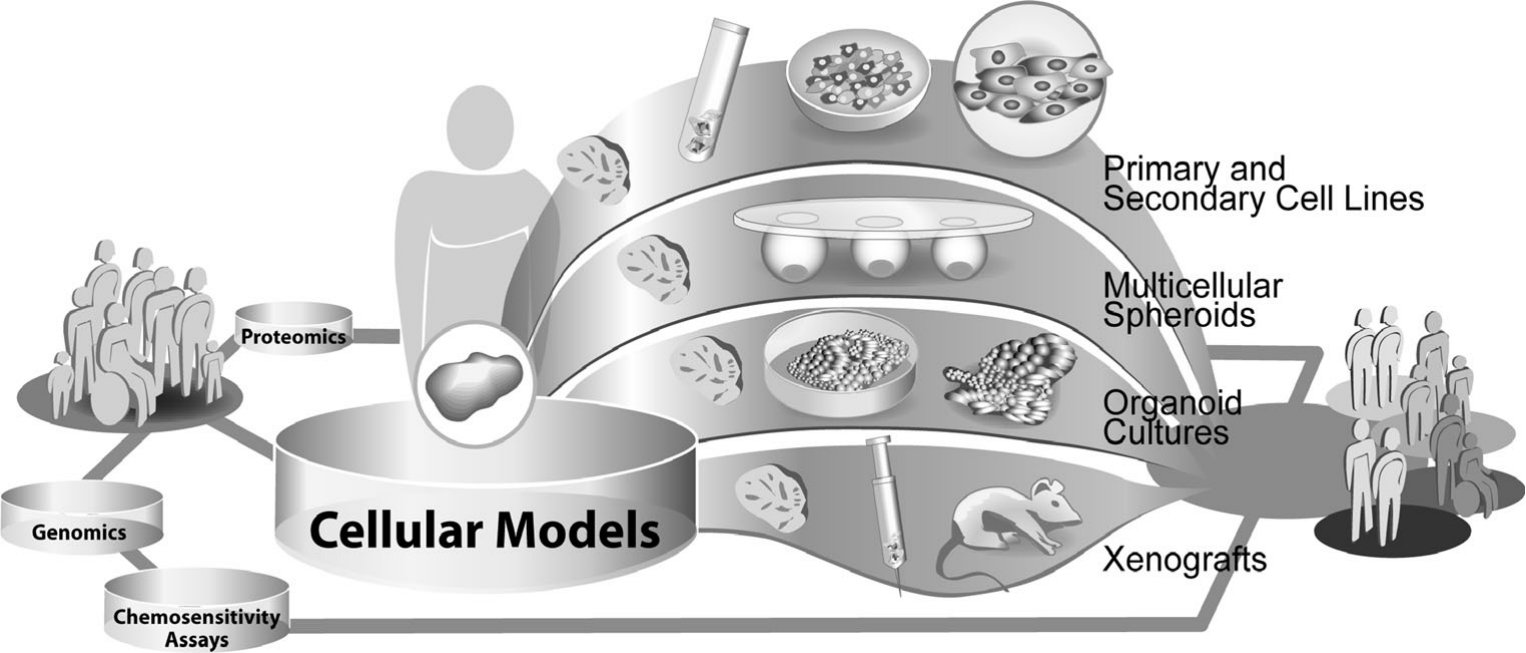
Exemplary illustration of different cellular models used in translational research

The prediction of response to chemotherapy at the molecular level is currently mostly based on data derived from in vitro experiments (Fig. 2). Besides studies in patient populations and xenograft models of tumors, cell cultures are the most commonly used in vitro systems for the analysis of cellular responses to drug treatment. Various types of cell culture models exist. These models differ in their ability to reflect the in vivo situation, which is of great importance for further translation of results to the clinical setting.

As a result of the gain in knowledge of cancer-specific signaling networks and metabolic pathways, it became obvious, that cell behavior is strongly influenced by the microenvironment of the cell [1, 2]. These findings had great impact on the development of in vitro cell culture models and their use in drug discovery and translational research. 2D cell cultures are the oldest and widely used models in cancer research, comprising mainly clonal-secondary and infrequently primary cell lines. Clonal-secondary cell lines are inexpensive in acquisition and easy to handle. Due to their ability to grow infinitely, they are well applicable in high-throughput screenings, suitable for genetic modification and good sources for preparations of cell components (e.g. mitochondrial-, membrane-, nuclear fractions). However, the preparation of cell lines from a tumor, results in loss of the 3D in vivo structure and in diversity of cell populations, thus these models only partly represent the origin tumor. Alongside the progress in laboratory technologies, the design of more and more extensive in vitro models became possible. Based on first attempts to rebuild 3D tumor structures, using secondary cell lines and natural as well as artificial extracellular matrices (ECM’s), the techniques for the preparation of such models rapidly advanced. Currently, the mixed culture of different cell types, the use of feeder layer cell lines and the induction of angiogenesis in these 3D cell culture systems are main improvements in this area of research. Nonetheless, these models represent artificial microenvironments and many features of an original tumor cannot yet be displayed. Complex models such as in vitro 3D-organoid cell cultures or xenografts currently best display the characteristics of an in vivo tumor. The cultivation of vital tumor tissue slices, for example, enables drug testing in a natural tumor environment and has the capability to reveal tissue composition dependent cellular responses to anticancer therapy. Xenografts also have the ability to mimic the in vivo microenvironment of a tumor in a physiological context, regarding nutrient supply, angiogenesis etc. However, using this model, differences in metabolism, body size and genetic background between the host species and humans have to be considered.

In summary, organoid cell cultures and xenografts represent valuable “bridge models” between in vitro cell lines and the clinical in vivo setting. The choice of a cell culture model for research should depend on the application in the study design and cost-benefit ratio.

### Primary and secondary cell lines

Over 60 years ago, the first human clonal cancer cell line was established from a patient’s tumors. Today, human tumor-derived clonal cell lines are able to grow in vitro, are easy to handle and thus they find wide application. Thousands of cell lines from diverse tumor entities can be purchased from different suppliers (e.g) [3, 4]. These cell lines are characterized and usually delivered including basic data, such as genetic profile (STR), morphology, doubling time, cytogenetics and references, by which additional data can be received using literature search. Being such robust and easy to handle models, secondary cell lines are a preferred starting point for the analysis of cellular mechanisms, e.g. resistance to anticancer therapy and signaling pathways. These models are also routinely used in versatile applications, e.g. testing of efficacy of compounds, examination of metastasis mechanisms, preparation of cellular compartments, extraction of proteins and DNA. Furthermore, secondary cell lines are well suited for artificial manipulation of cell characteristics, such as expression of mRNAs and proteins, mutations (knock-in) and modulation of chemosensitivity.

For example, approaches to understand acquired drug resistance are cancer cell lines with established drug resistance. Continuous exposure of these drug-sensitive cell lines to anticancer therapeutics in vitro, selects for the relatively rare drug-resistant clones, which are then further raised to a chemoresistant sub clone cell line. Comparative analysis of properties of the parental drug-sensitive cell lines and the selected drug-resistant cell lines has the potential to identify specific molecular mechanisms of drug resistance [5]. Hence, transformed cell lines and their parental counterparts are also commercially available and represent artificial, but defined models for the investigation of determinants of chemosensitivity.

Nowadays, secondary cell lines are integrated in huge compound screening programs for drug discovery and research programs to understand the underlying mechanism of individual response to chemotherapy. Secondary cell lines fulfill all requirements for implementation in high-throughput screenings, enabling the rapid screening of large panels of compounds. The National Cancer Institute 60 (NCI60) platform was the first high-throughput cancer cell line screening program and therefore triggered the development of adequate techniques. The experimental methods had to be adapted to the requirements of economic, high-throughput screenings, e.g. high-content data mining, automation of handling liquids, miniaturization of cell culturing and drug testing procedures. A major finding of the program was that compounds with similar patterns of cell line chemosensitivity tend to have common mechanism of action, which led to the development of new algorithms for data analysis and adaption of study designs. The NCI60 anticancer drug discovery program was reviewed in detail by Shoemaker [6], who highlighted its history and methodology. Learning from the NCI60 experiences, the Cancer Chemotherapy Center of the Japanese Foundation for Cancer Research (JFCR) established the JFCR-39 platform. This panel of 39 human tumor-derived cell lines included a subset of the NCI60 cell lines and additional gastric cancer cell lines [7]. A new algorithm for data analysis enabled the comparison of newly screened compounds with previously screened compounds to discriminate between new or previously described modes of action. Using the COMPARE algorithm and advanced data mining techniques, several new anticancer agents [8–11] were identified.

In drug discovery or predictive biomarker studies for the introduced ‘targeted anticancer therapeutics’, small panels of cancer cell lines cannot display the clinical activities of these compounds, which are often limited to small subgroups of molecular-defined patients. Taking this into account, high-throughput screenings are now being adapted to much larger panels of cell lines. To capture the genetic heterogeneity among diverse cancers, Mc Dermott and colleagues [12] developed an automated platform for the screening of the chemosensitivity of 500 solid cancer cell lines to kinase inhibitors. In this study, they observed the expected response rates with only small subgroups of cell lines showing responses to particular compounds. Therefore, a comprehensive cancer cell line platform was established, currently including 1,200 cancer cell lines. Due to the fact that only around 80 % of those secondary cancer cell lines are adaptable to high-throughput screening, mostly caused by technical limitations such as insufficient doubling times or atypical culture requirements, this panel is referred to as the Center for molecular Therapeutics 1000 (CmT1000) [13]. This cell line panel is currently being used to investigate the genetic determinants for chemosensitivity. First results from this large data sets showed that tumor-derived cell lines recapitulate clinical findings concerning responses to targeted inhibitors [14].

Another, very recent approach in generating primary cell lines for in vitro experiments has been introduced by Lui et al. [15]. This approach initially comprised a method to indefinitely extend the life span of primary human keratinocytes using both fibroblast feeder cells and a Rho-associated kinase (ROCK) inhibitor, and is also efficiently applicable to establish cell cultures from human and rodent tumors. This innovative technique provides significant opportunities for cellular diagnostics and molecular therapeutics (drug profiling), expands the value of biobanking and has the potential to greatly improve personalized medicine.

A general disadvantage of secondary cell lines is that they only represent one cell from a diverse tumor microenvironment which resembles the capabilities necessary for adapting to in vitro culture. It is still unclear in which manner adaption to in vitro culturing and multiple passaging influences cell characteristic/behavior. The establishment and cultivation of primary mixed single cell cultures always have been quite complicated [16]. Primary mixed cell cultures isolated from patient’s tumors represent a wide spectrum of cell types abundant in vivo. This diverse mixture mainly consists of different epithelial- and mesenchymal cancer cells, tumor associated stroma and immune cells [17, 18]. Therefore, these primary cell cultures more closely reflect the in vivo situation than secondary, clonal cell lines. However, several difficulties are still to overcome, while establishing primary mixed cultures. The basis for the preparation of primary, mixed cell cultures is vital tumor tissue and experience in cell culture handling. Besides the quality of tumor tissue, the method for preparation of single cells from a tumor, the surface preparation of cell culture dishes and finally the composition of the culture media are also essential parameters for a successful establishment of primary mixed cultures. The artificial shifts in and losses of cell populations, due to unnatural in vitro culturing and passaging, limits the maximal diversity of cell types to low passage primary, mixed cultures. Most studies using primary cells prepare cell cultures shortly after tumor resection and disseminate cells directly for experiments. Studies regarding the in vitro chemosensitivity of primary cells were conducted in different tumor entities e.g. small cell lung cancer [19], colorectal cancer [20, 21], gastric cancer [22], Leukemia [23, 24], ovarian cancer [25–27] and head and neck cancer [28, 29]. One limiting factor is that, the diversity of cell types will decrease during in vitro cultivation, due to the dissimilar ability of different cell types to proliferate in vitro and survive passaging. Another issue limiting the predictive value of these cell cultures is the loss of the 3D architecture of the origin tumor. Although the in vitro analysis of cultured cell lines is associated with artifacts related to effects attributed to a non-physiological environment and long-term passage in culture, it was shown that cancer cell lines retain most of the genomic features of the primary tumor [30, 31]. This has not yet been shown for proteomic features of cancer cell lines. The awareness of the importance of the tumor microenvironment and the three-dimensional aspects of solid tumors, in the response to anticancer therapy has initiated efforts to display these features in vitro more accurately [32–34].

There are also several other important factors to take in regard to mimic the in vivo microenvironment of a tumor in vitro. For example, a whole field within cancer research is dedicated to the investigation of hypoxia, which is defined as inadequate oxygen supply to cells and tissues, in solid tumors and implications on anticancer treatment [35–40]. The oxygen concentration of 21 %, used in most in vitro culture systems is not physiological in regard to the limited oxygen supply of cells within a solid tumor.

### Multicellular spheroids

Since it has been shown that the cellular signaling network, e.g. regulation of apoptosis is influenced by 3D cell organization and multicellular complexity, new cell culture models for a more realistic investigation of tumor cell behavior ex vivo are urgently needed [41]. To establish such models, it is necessary to maintain or reconstitute an environment which closely resembles the tumor in vivo. One of the first approaches of rebuilding the 3D microenvironment during in vitro cultivation and drug testing was the development of a culture model called “Spheroids”. In 1970, the first spheroid model was devised by Sutherland [42]. Meanwhile, spheroids have been grown from a variety of normal and tumor cell lines and used in different assays, to study anticancer therapy efficiency as well as 3D cellular interactions [43, 44]. Single cell cultures were used to establish an organoid-like 3D model using different techniques [45, 46]. These different culture techniques include various artificial as well as natural ECM`s [47, 48] and mechanical methods to generate defined, roundly shaped cell clusters. Matrices, such as agarose, collagen, gelatin or matrigel allow the establishment of culture systems with well-defined geometry, wherein the 3D structure affects interactions between cells. This usage of 3D matrices has been reported to show fruitful results in recapitulating tissue functions in 3D [49, 50]. Besides various cancer cell lines, cell types like Madin–Darby canine kidney cells and fibroblasts, have also been monitored in 3D contexts and have provided valuable insight into the basic molecular mechanisms of polarity, adhesion, cell migration and response to anticancer therapy [51–53]. Numerous studies have documented differences in cancer drug sensitivity between cells cultured in monolayers and those grown in 3D cultures [54–56]. Previous studies have shown that certain drugs are more effective in 3D cell culture systems [57–60], although other drugs showed greater activity in the 2D cell culture systems [61, 62]. These days, fewer than 100 human tumor cell lines have been reported to grow in spheroid cultures [63]. Platforms based on tumor spheroids have been developed and are being used for analysis of individual chemosensitivity and secondary screening of potential new anticancer compounds [64, 65]. The application of spheroids in drug screenings has been reviewed by Friedrich and colleagues [66]. However, it remains to be demonstrated comprehensively that chemosensitivity data derived from 3D cell cultures captures clinically relevant responses more precisely than standard 2D cultures. Furthermore, these systems cannot completely mimic the complex tissue architecture and the high degree of variability seen in individual tumors.

### Organoid cultures

It has been shown that signaling and metabolic pathways in cell lines have distinctly different expression patterns compared to tumor tissues. Pathways in cell lines tended to be upregulated compared to tumor tissue with exceptions in genes involved cell adhesion, ECM-receptor interaction and focal adhesion [34, 67]. As discussed before, spheroids are a good approximation to the in vivo tumor, but still lack the natural tumor environment, including the state of receptors and corresponding extracellular signaling between diverse cell types naturally being present in the tumor. Therefore, the development of in vitro organoid cell culture models was an essential step for translational research. First experiments were performed in 1967 by Matoska and Stricker, using tumor cubes of approximately 1 mm^3^ [68] for in vitro culturing. Later, an in vitro histoculture system, using a native-state collagen-sponge gel to support the three-dimensional growth of tumor tissue sections was developed, called the Histoculture Drug-Response Assay (HDRA) [69]. Features of the histoculture system include the maintenance of three-dimensional tissue architecture and the use of histological autoradiography or colorimetric assays as endpoints for determination of chemosensitivity [70, 71]. Ohie et al. [72] published a protocol on the Method of the HDRA. The reliability and utility of the HDRA were examined in several clinical studies for different tumor entities, e.g. oral squamous cell carcinoma [73], head and neck cancer [74], gastric cancer [75], colorectal cancer [76] and ovarian cancer [77]. Up to now, it has not been shown that the HDRA is also able to predict efficiency of targeted drugs such as small molecules and antibodies.

The past years have seen unprecedented developments in the use of human tissue surrogates in vitro. Clevers et al. [78] developed a technique in which adult stem cells, originating from fresh tumor tissues, are embedded in a three-dimensional matrix and allowed to self-organize into epithelia of the respective organ of origin. The resulting organoids represent the physiology of native epithelia much better than traditional cell lines. Mini-guts, for example, reproduce the epithelial architecture of small intestine and colon [79, 80]. If combined with genetic information and pharmacological profiles, such an organoids could aid in identifying markers that predict a patient’s drug response similar to the Cancer Cell Line Encyclopedia [81].

Parallel to the development of tissue microtomes enabling the preparation of thin slices of fresh tissue, precision cut cancer tissue slices from tumor tissue have become more popular as ex vivo systems. It has been shown, that cell viability of tissue slices was maintained in in vitro culture for at least 4 days [82]. After treatment with different compounds (chemotherapeutics, small molecules, antibodies), slices can be fixed by immediate freezing or by formalin. Frozen slices can be used for several assays, e.g., functional drug effects on viability (ATP), apoptosis (activation of caspase 3/7), proliferation (BrdU) and signal pathway analysis (activation of phosphoproteins). Formalin-fixed slices can be utilized for immunohistochemical analysis of target expression, drug effects and cell–cell interactions. Furthermore, laser capture micro dissection can be applied, allowing the separation of different cellular compartments, for molecular analysis of pure cell populations. Viara and colleagues reported on a preclinical model of organotypic culture for pharmacodynamic profiling of human tumors [83]. This model demonstrates the ability to detect pharmacological interventions ex vivo in a presevered original cancer microenvironment. Due to the broad spectrum of molecular techniques that can be implemented, organoid cell culture models offer a unique opportunity to understand the complex basis of cellular responses to anticancer therapeutics of all groups, e.g. classical chemotherapeutics, small molecules and therapeutic antibodies [84]. Despite the advantages of the models, difficulties in obtaining specimen and limited viability of these tissues in culture over time represent major obstacles. The successful cultivation of tissue slices is also dependent on tumor entity, highly adapted culture conditions in terms of media supplements and other culture techniques. In the future, the use of miniaturized cell-based models that are specifically engineered to closely reflect in vivo behavior can reduce costs and add efficiencies to drug development, but most importantly increase the accuracy of molecular prediction of response to anticancer therapy.

### Xenografts

Currently existing in vitro cancer cell culture models, such as primary cell lines and organoid cultures are a solid basis for molecular drug testing, but they do not reflect the natural tumor environment in all facets. The final application of anticancer drugs takes place in the in vivo situation, in the patients. Since it is unethical to use patients for preclinical research, xenograft cancer cell culture models were developed to facilitate drug testing in vivo and thus improve basic and translational research and prediction of individual response to chemotherapy. Cancer cell characteristics, such as chemosensitivity to anticancer chemotherapy, are strongly affected by several parameters in a physiological, in vivo, situation. In contrast to in vitro cell culture models, xenograft models offer micro environmental conditions, e.g. tumor architecture, angiogenesis, metastasis close to the real patient. The injection of vital human cancer cells or even transplantation of human tumor fragments is therefore still essential to study cancer in an in vivo situation [85, 86]. Among the existing in vivo cell culture models, the mouse model is widely used. It bears the relative advantages of good availability, low space requirements, low cost, ease of handling and fast reproduction rate. Mouse xenograft models are extensively being used to study individual response to anticancer therapy and drug development [87, 88]. Several studies on DNA and protein level were conducted in mice xenografts to understand and predict response to anticancer therapy. For example, gene expression signatures and plasma protein biomarker have been reported to predict efficiency of therapy ex vivo [89–91].

But there are also multifaceted parameters affecting outcome when conducting xenograft experiments, e.g. site of implantation, growth properties and size of tumor at the time treatment is administered, agent formulation, scheduling, dose and the selected endpoint for assessing activity. A basic review on the mouse model in drug testing was published by Mattern L. and colleagues in 1988 [92]. The application of xenografts in drug testing has been reviewed elsewhere in detail [93, 94]. Despite the relatively comprehensive ability of mice models to mimic the clinical situation in patients, there are differences between mice and humans which might have an impact on the predictive value of this model [95]. Mice and humans obviously differ largely in body size and lifespan. Although mice have a similar incidence of cancer at the end of the life cycle, they primarily develop cancers in mesenchymal tissues, e.g. lymphomas and sarcomas. Most cancers in humans are of epithelial-origin and lead to carcinomas. Furthermore, the basal metabolic rate of mice is much higher, which results in increased generation of reactive oxygen species, other mutagens and also distinct metabolism of anticancer drugs in mice from humans.

Xenografts may also fail to recapitulate immunological aspects of tumor-stroma interactions that are present in human patients. Cell signaling interactions between cancer cells and host stromal cells may not occur properly due to interspecies incompatibilities, e.g. interactions of ligands of one species with receptors of the other [96, 99]. Those incompatibilities may impact various characteristics of tumors, e.g. drug response and metastatic behavior. [97, 100]. A short overview of the challenges of selecting the ‘right’ in vivo oncology pharmacology model and improving the translation of these models to a clinical setting was summarized by Firestone B, 2010 [98, 101].

Nonetheless, xenograft model are useful preclinical models. The better these models are characterized on genome and proteome level and by implementing the learning experience while using these models, the more basic information on the individual response to anticancer therapy will be gained.

## Chemosensitivity assays

**Fig. 3:**
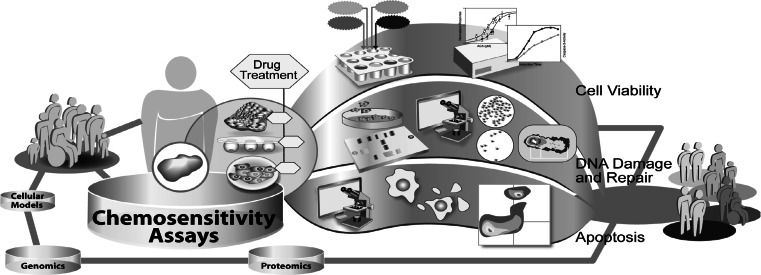
Exemplary illustration of different chemosensitivity assays used in translational research

First experiments to determine the individual chemosensitivity of tumor cells from cancer patients were made in the mid-1950’s [102]. At that time, techniques for chemosensitivity testing were developed on the basis of well-known parameters such as colony forming ability, growth inhibition or cell viability. In theory the overall effects of cytotoxicity are the sum of all specific cellular effects underlying multi-factorial mechanisms. Therefore, in vitro chemosensitivity testing can potentially predict response to anticancer therapy either by determination of the death of all cancer cells or at least by complete growth inhibition. Currently, chemosensitivity tests find wide application in basic and translational research (Fig. 3). The measurement of drug effects on cell viability is integrated in basic research, for the detailed analysis of efficiency and mode of action of drug candidates, as well as in the clinical setting for the general determination of chemoresistance of a patient’s tumor. Firstly, the measurement of cancer cell chemosensitivity to miscellaneous compounds with potential anticancer activity is the basis of most drug discovery programs. Previous publications described various phases of the development of an in vitro anticancer drug screen, aimed at the identification of compounds showing selective growth inhibition or cytotoxicity towards particular cell or tumor types [102, 103]. These screening programs require very robust, automated chemosensitivity assays for the measurement of drug effects on cancer cell viability or growth. Therefore, many studies were performed comparing chemosensitivity assays in regard to their sensitivity, reproducibility, applicability to cancer cell lines of various origins and potential for adaption to high-throughput [24, 104–106]. Secondly, in vitro chemosensitivity tests are, to some extent, applied in the clinical setting to determine chemoresistance in a patients` tumor. This may help to guide individualized anticancer therapy, especially in second-line treatment where the guidelines for therapy are not always clearly defined [107].

In vitro chemosensitivity tests are not approved for predicting or guiding therapeutic treatment of patients in first-line therapy or routine use.

### Implementation of cell viability assays in preclinical drug testing

Besides a whole spectrum of assays, measuring events indicating cell viability, the most often used cell viability tests today in chemosensitivity testing are the MTT assay [108], the FMC assay [109], the ATP-TCA [26, 110] and SRB assay [102]. The four different assays measure cytotoxicity as a decrease of fundamental metabolic activity (MTT assay, FMC assay) or by the reduction of essential biomolecules (ATP assay) and cell mass (SRB assay). The MTT tetrazolium salt colorimetric assay is based on the metabolic reduction of 3-(4, 5-dimethylthiazol-2-yl)-2, 5-diphenyltetrazolium bromide (MTT). The yellow tetrazolium salt MTT is converted by mitochondrial dehydrogenases of metabolically active cells to an insoluble purple formazan product. The optical density can be detected by precise spectrophotometric measurement using a plate reader [111]. The fluorometric microculture cytotoxicity assay (FMCA) measures fluorescence generated from cellular hydrolysis of fluorescein diacetate (FDA) to fluorescein by cytosolic esterase activity. The measured enzyme activity in combination with indirect detection of cell membrane damage is determined as parameters for cell viability [112]. In the ATP-tumor chemosensitivity assay (ATP assay) the intracellular ATP content is quantified by measuring luminescence produced by a reaction of ATP with luciferase and D-luciferin [113]. The assay allows for a rapid, sensitive measurement of cellular ATP content. ATP levels are linearly related to the number of viable cells and increased with time in cell line cultures correlating with growth kinetics [114]. The sulforhodamine B (SRB assay) measures whole protein content for the detection of viable cells. SRB is a pink aminoxanthene dye with two sulfonic groups that bind to basic amino-acid residues under mild acidic conditions. The binding of SRB is stoichiometric, the amount of dye extracted from stained cells is directly proportional to cell mass [27]. Using this assay to determine cell growth or viability, one assumes that dead cells either lyze, are removed during the procedure or otherwise do not contribute to the colorimetric end point. After fixation and staining procedures, tested cells can be stored indefinitely, which also contributes to high-throughput applicability. [115, 116]. The quantitative results of these different chemosensitivity tests are similar, although sensitivity varies. The lowest sensitivity was found for the MTT assay. Here, a great number of cells (25,000 cells/well) are needed to get reliable results. The MTT assay is therefore not applicable when the tumor biopsy is small. For the SRB assay [117] and the FMC assay [27] around 2,000 cells/well are sufficient. The ATP assay is reported to be able to detect down to ten cells/well. In the past chemosensitivity assays have been technically optimized continuously. Several new data analysis methods were established, but the best comparison with the clinical outcome has yet been achieved by the “sensitivity index” (SI) rather than the determination of the IC50 values or the AUC index [118]. Currently, chemosensitivity is a basic parameter for anticancer drug efficiency and in combination with several other read outs of drug effects integrated in several drug discovery and preclinical drug testing platforms.

For the initial large-scale drug screening program, called the in vitro anticancer drug discovery project of the National Cancer Institute (NCI), the sulforhodamine B (SRB) assay has been chosen, because of its high level of sensitivity, adaptability to high-throughput screening and endpoint stability [115, 116]. This project tested 10,000 or more samples per year in a manner that requires robust technology for the analysis of several million individual measuring points [119]. The SRB assay has mostly been used for the measurement of cytotoxicity and cell growth in high-throughput screening and basic research. Thus, in vitro chemosensitivity data from this assay has rarely been correlated to clinical outcome. By contrast, the chemosensitivity data from cancer cells measured with the MTT, FMC and ATP assays have been correlated with the efficiency of anticancer therapy in a clinical setting. Thereby, the ATP assay was preferably applied. Even though not all cancer types can be examined because of the above described limitations, correlations of the in vitro data with clinical outcome were obtained for ovarian carcinomas [118], breast carcinomas [119], leukemia [120], melanomas [107], colorectal carcinomas [121], lung carcinomas [122] and gastric cancer [123]. Most results exist for ovarian and breast carcinomas.

Besides monotherapies, two or more anticancer drugs are often used in the in vivo clinical setting, sometimes applied simultaneously in other cases sequentially, with intermissions of one or more weeks. This situation can hardly be mimicked by in vitro assays. Without the knowledge of the pharmacokinetics of the single therapeutics applied in combination, the ratio of the substances in the cells is unknown. Strong concentration-dependent combination effects between different anticancer drugs were observed [124, 125]. Synergistic, as well as antagonistic effects were found depending on the sequence of drug treatment for the combination of paclitaxel and cisplatinum [125, 126] and for combinations of platinum compounds with paclitaxel and colchicines [127]. Besides these limitations another point of concern is the drug treatment time in vitro. During a short one-day incubation time growth inhibition or colony forming ability cannot be measured. An incubation time which allows the cells to duplicate, at least 2 days to several weeks, is necessary for the measurement of colony forming ability or growth inhibition. Therefore the duplication time of the cancer cells limits these methods. Furthermore, isolated primary cancer cell cultures are difficult to cultivate in vitro. The rate for successful cultivation and passaging of primary cell cultures is low. Stromal cells, such as fibroblasts which cannot be totally separated during isolation generally grow faster than the cancer cells, which may lead to false results. Serum free medium is reported to selectively reduce the growth of fibroblasts [128]. Soft agar used in clonogenic assays is also reported to hinder fibroblasts from forming colonies [129]. However, it is not known how these adapted cell culture conditions influence the growth and characteristics of cancer cells. Especially in cancer types where stromal cells influence cancer cell growth, e.g. squamous cell carcinomas the in vitro data may not reflect the in vivo situation.

The low response following physician choice especially in the second or third line therapy, with little if any benefit for the patients, demands in vitro chemosensitivity testing which leads to higher response rates as published previously [128]. In addition, for the determination of drug resistances these assays have shown convincing results [27, 130], which recommend the in vitro drug resistance measurement routinely at least in the second-line therapy. In the first-line therapy where the oncologist can choose between several chemotherapies with equivalently efficacious response rates, a chemosensitivity assay directed treatment could also be of advantage [128]. Several studies reported weak to good correlations of in vitro to in vivo data. Nonetheless, further studies were recommended for clinical validation by most authors. The American Society of Clinical Oncology (ASCO) furthermore recommended comparing patients, whose individual therapy resulted from chemosensitivity testing with patients, whose therapy was chosen empirically [131]. They did not recommend in vitro chemosensitivity testing for chemotherapy guidance outside of clinical trials. Others contradicted the ASCO especially because of the low number of studies considered in their review article leading to insufficient conclusions [132, 133].

In summary, chemosensititvity testing is deeply integrated in basic drug testing and preclinical research. Various methods are used to determine the sum of various specific and unspecific drug effects on cells as decrease in viability or cell death. The application of individualized anticancer therapy based on in vitro chemosensitivity testing in the clinical setting has been conducted using several different laboratory methods [96, 134]. Correlations of in vitro results with clinical outcome have indicated predictive accuracies of 57–83 % for drug sensitivity and >90 % for drug resistance [135–139]. Although studies have demonstrated the predictive value of different chemosensitivity assays, the insufficient number of prospective randomized studies validating efficiency and benefit has yet limited the routine application in the clinical setting.

### DNA damage and repair

Other multi-factorial endpoints such as DNA damage [140] and DNA repair [141] were examined as parameters for determining cancer cell chemosensitivity, as well. A broad spectrum of anticancer drugs induces DNA damage which, in turn, leads to cell death. Quantitative DNA damages could therefore correlate with the clinical outcome of patients treated with DNA damaging chemotherapeutics. The comet assay, a method for the measurement of DNA damage [140, 142] is used in chemosensitivity testing because it is a quick, sensitive method that does not require cell division. Also, only very few cells are needed, so that cell numbers obtained by needle biopsies are in general sufficient. Unger et al. [143] measured DNA damage induced by cis- and carboplatin, doxorubicin and gemcitabin in primary cells of ovarian carcinomas. In parallel, they measured the cell chemosensitivity and correlated both parameters. Like others, they found a strong correlation for the platinum compounds [144] but not for doxorubicin and gemcitabin. Multiple targets of these cytostatics may explain these results. For platinum compounds the main cause of cytotoxicity is thought to be the induced DNA damage [145]. In the future the comet assay could become important in testing for platinum resistances in patients especially in the second-line and third-line therapies.

DNA damage is coupled with DNA repair, which has been reported to be heterogeneous among individuals [146]. For example, the determination of an individual degree of induced DNA damage and repair capacity using the comet assay [147] is therefore thought to be a prognostic factor for chemosensitivity. Furthermore, DNA damage response pathways have been shown in experimental models to be associated with resistance or sensitivity to DNA damaging agents. Teodoridis et al. [148] examined potential associations of methylation patterns of DNA damage response genes with response to anticancer therapy. Over the last decade, there has been a tremendous increase in the understanding of the mechanisms of DNA damage detection, signaling, and repair, and these findings have suggested therapeutic opportunities for anticancer drugs that modulate these pathways [149, 150]. For example, there is a large body of experimental evidence showing that DNA damage checkpoint kinase inhibitors can enhance the efficiency of both conventional chemotherapy and radiotherapy, and several agents have entered clinical trials [151]. In the presence of DNA lesions, cell cycle checkpoints and repair mechanisms are being activated and a prominent route of cell elimination is apoptosis [152]. Specific DNA lesions induced by DNA damaging anticancer drugs that trigger apoptosis have been identified. These include O6-methylguanine, base N-alkylations, bulky DNA adducts, DNA cross-links and DNA double-strand breaks (DSBs). DNA damage induced cell death by apoptosis has been reviewed by Roos et al. [153].

### Apoptosis

In 1972, Kerr, Wyllie and Horvitz described the phenomenon of programed cell death and initially called this process of natural cell death apoptosis. Since then, apoptosis has developed into an area of intense scientific interest which encompasses the study of mechanisms involved in mediating the cell biology of programed cell death. Two major cell-intrinsic pathways for inducing apoptosis have been identified. One begins with ligation of cell death receptors, and the other involves mitochondrial release for cytochrome c. Both pathways result in characteristic morphological changes in nearly all cell types such as membrane blebbing cell shrinkage, nuclear fragmentation, chromatin condensation and chromosomal DNA fragmentation. Many of the changes reflect the selective proteolytic cleavage of various intracellular polypeptides (e.g., lamins, caspases). Based on these alterations many different in vitro methods have been devised to detect apoptosis. Examples are the TUNEL (TdT-mediated dUTP Nick-End Labeling) analysis [154], the DNA laddering analysis for the detection of fragmentation of DNA in populations of cells or in individual cells [155], the Annexin-V analysis that measures alterations in plasma membranes [156, 157], and the activation of caspases (family of cysteine proteases) [158]. In addition, apoptosis related proteins such as p53, Fas, Bcl-2 and Bax are commonly analyzed to understand details of the complex picture of the apoptotic pathways.

Previous studies have demonstrated that a wide range of anticancer agents, including chemotherapeutic agents, hormones, and various biologicals, induce apoptosis in malignant cells in vitro. Since apoptosis is a regulated process, biochemical alterations that make cells more or less susceptible to apoptosis might affect their sensitivity. It has been proposed that tumor chemosensitivity to anticancer drugs may partly be attributable to the degree of activation of a genetic program for cell death. One of the current models suggests that many different anticancer drugs such as doxorubicin, etoposide, and cisplatin trigger apoptosis by inducing the synthesis of FasL, which ligates Fas and activates caspase-8. However, other studies have revealed many exceptions to this model and propose that the majority of anticancer drugs initiate apoptosis by the cytochrome c/Apaf-1/caspase-9 pathway resulting in mitochondrial membrane permeabilization (MMP). Since mitochondrial permeabilization is a relatively early event in the apoptosis, detecting this event might be more useful in revealing the presence of apoptotic cells than other assays, such as those that measure caspase-3 activation or DNA fragmentation. In vivo studies of induction of apoptosis in experimental models and patients undergoing therapy have yet been limited to histological examination, thus providing a static picture of apoptosis, rather than an observation of ongoing cell death. However, in vivo detection of apoptosis is also hampered by the rapid clearance of apoptotic cells by phagocytes [159]. Preclinical studies on tumor cells analyzing the contribution of caspases to anticancer therapy resistance have to our knowledge not yet been published. However, inhibition or loss of caspase expression has been proposed to confer resistance to different anticancer drugs [160, 161]. The potential impact of caspases and their activators on resistance is also supported by ‘knock-out’ mouse models with distinct variations of the Apaf1, caspase-3 or caspase-9 gene, which are resistant to various apoptotic stimuli in different tissues [162–164].

Finally, alterations in the p53 gene and implications in the induction of apoptosis represent one of the most studied genetic events in cancer cells and are suggested to be linked to chemosensitivity. Nevertheless contradicting results were reported. For example, significant correlations of overexpression of p53 and prognosis were published [165] for squamous cell carcinomas, but no correlations were found in another study [166].

Detailed analysis of the induction of apoptosis is nowadays mainly integrated in comprehensive drug testing platforms for preclinical testing of anticancer drug candidates. Therein, the in vitro analysis of the mode of action and efficiency of a drug candidate is the main focus.

## Genomics

**Fig. 4:**
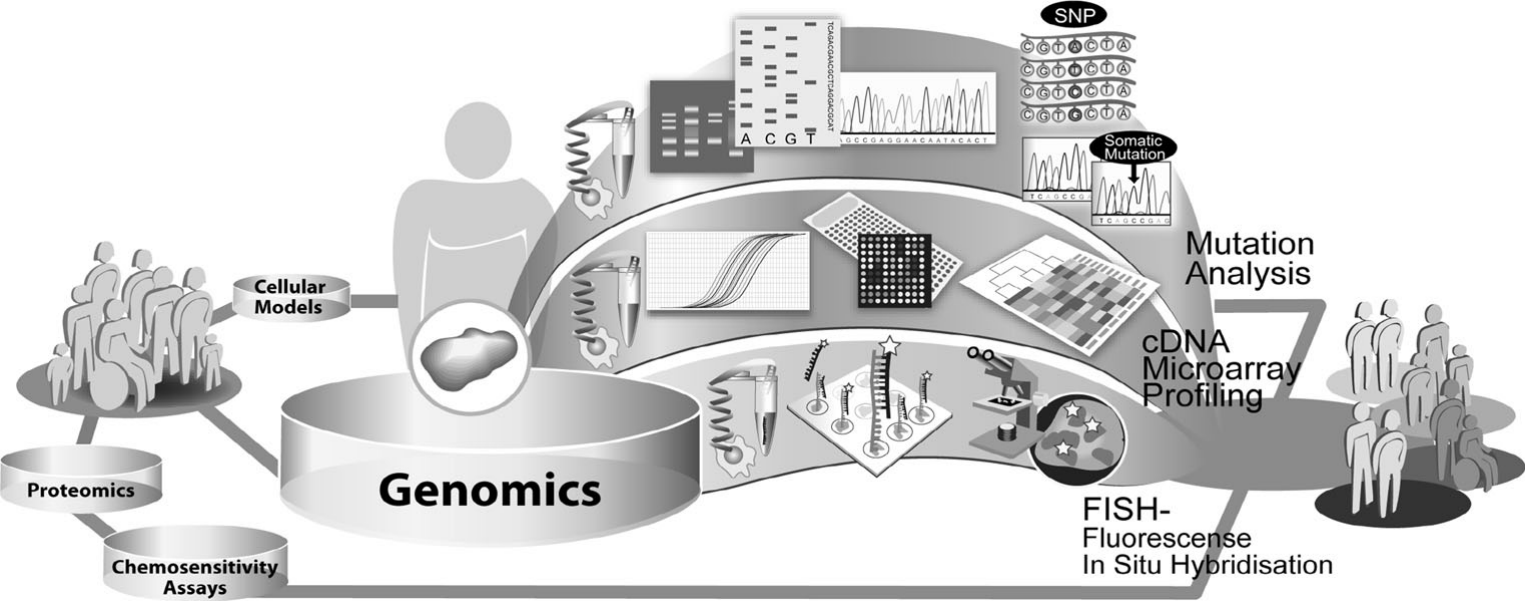
Exemplary illustration of different genomic approaches used in translational research

Cancers arise from a multistage process in which tumor cells progressively acquire a sequential accumulation of genetic alterations. The genomic changes occurring in the transformation of normal cells to cancer cells influence several genetic mechanisms. These events destabilize the normal cellular homeostasis e.g. gains, losses or translocations of large regions of chromosomes, single-nucleotide substitutions, copy number changes and methylation events. Among these alterations, intragenic mutations play an important role in activating oncogenes or inactivating tumor suppressor genes. This results in misregulation of cellular signaling, e.g. proliferation and apoptosis and thus generates a survival advantage for the cancer cell [167, 168]. Certain mutations may be associated with specific types of cancers or may be common to several types of cancers. Currently, most of our knowledge of these alterations stems from studies of single genes in specific cancers. A variety of high-throughput techniques has now been developed for profiling and analysis of cellular networks, providing means to survey the cancer genome and transcriptome. By the complete sequencing of the human genome and progress in bioinformatical research, the understanding of cancer-related genomic alterations and expression patterns has grown. Nonetheless, understanding of the complex basic patterns and functions of molecular alterations on the genomic level are a great challenge. Therefore, the main focus of oncogenomic profiling lies on the analysis of DNA repair, mutation status, gene expression, gene copy number and genome stability (Fig. 4). Based on the current knowledge of the oncogenomic alterations existing in cancer genotypes, key mutations of cancer development [169], new subclasses of cancer types have been identified [170, 171] and even models for the prediction of clinical outcome have been calculated [172, 173].

The prediction of response to therapy is a relatively new field of oncogenomics. The strong heterogeneity of individual tumors in terms of clinically observed drug response is an important reason for the need for individualized, molecular guided therapy, but also for the difficulties in realizing this goal. There is accumulating evidence that drug-specific response pathways are influenced by the individual genotype and gene expression, which led to efforts to identify gene signatures and gene mutations predictive for chemosensitivity. This will ultimately lead to the development of strategies for patient-tailored anticancer therapy which are based on the individual molecular profile of a tumor [172].

### Mutation analysis

During the past decades, there have been great advances in experimental methods for genome characterization built on ‘first-generation’ capillary-based DNA sequencing, also known as Sanger sequencing [173]. These sequencing methods can be crudely divided into a general and a targeted approach. The products of both approaches are amplified templates, either by multiplication in plasmids or as PCR amplicons. Sequence determination is then performed by high-resolution electrophoretic separation in a capillary-based polymer gel. Bioinformatical software converts these signals into DNA sequences and calculates error probabilities for each base-call. Approaches, like the second-generation parallel sequencing techniques increased the throughput and decreased the cost of nucleotide resolution. Second-generation sequencing technologies are based on the simultaneous detection of nucleotides in arrayed amplified DNA products originating from single DNA molecules [174]. Advanced technologies like next-generation sequencing approaches are currently at the front of research with great potentials to give new insights in tumor heterogeneity and individual drug responses. High-throughput sequencing technologies including those created by Illumina (Illumina, Inc.), 454 (Roche Diagnostics Corp.) and SOLiD (Life Technologies), enable whole genome sequencing at an unprecedented scale and dramatically reduced costs over the gel capillary technology used in the human genome project.

Advanced next-generation sequencing systems are capable of sequencing a human genome at 30× coverage in less than 1 week. These next-generation sequencing (NGS) systems use parallel sequencing to generate hundreds of millions of short (36- to 150-bp) DNA reads that can be aligned to the human genome. Although a number of different NGS strategies have been developed, the paired-end strategy from Illumina Inc. has become the tool of choice for most cancer genome studies published to date. While most cancer genome studies so far have focused on single patients, this pattern is changing as a result of ongoing international collaborations and decreases in the cost of sequencing. The hope is that NGS data will shorten the road to personalized medicine, in which treatments and therapies are tailored to target the unique features of individual tumors and tumor subpopulations [175] based on mutations that define sensitivity and drug resistance (http://www.cancerrxgene.org/).

The extensive genotyping of individual tumors displayed thousands of mutations in an individual cancer genome [176]. The extent of genetic variation in the genomes of the human population is far greater than had been estimated [177]. While the impact of the vast majority of these mutations currently remains unknown, basic and translational research has pointed out, that a much smaller group of mutations is not only necessary for the development of cancer but is also required for the maintenance of the tumors´ survival [178]. The presence of such ‘driver’ mutations sustains tumors and can simultaneously represent a cancer-specific target for therapy [179–182]. For example, the mutations of the tumor suppressor genes TP53 and EGFR are among of the most studied mutations in cancer research with implications in tumor development, progression and response to chemotherapy [183]. Several mutations in genes, encoding for proteins involved in cell signaling pathways, have a strong impact on the field of targeted therapy. This area of cancer research is reviewed elsewhere in detail [180, 184–190].

Individual alterations and differences in metabolism, in- and efflux of xenobiotics and cellular signaling pathways caused by mutations are some of the reasons for the diversity in individual response to conventional chemotherapy [191]. The most common form of mutation in the human genome is the single-nucleotide polymorphism (SNP). Functional genomic polymorphisms in drug target genes [193], metabolising enzymes [194] and DNA-repair enzymes [195] may have important implications for drug efficiency. Therefore, these sequence alterations are determinants of variations in metabolism of drugs and associated side effects, because they have an important influence on the expression levels and activities of the corresponding proteins [196]. A recent study found that anticancer drug susceptibility-associated SNPs were associated with the transcriptional expression level of genes as potential master regulators [197]. A significant body of evidence supports the concept of predicting drug efficiency and side toxicity by SNP genotyping. For example, transcriptional contributions of genetic polymorphisms to cytotoxicity of cisplatin using human cell lines were listed [198]. Furthermore, in 2010 a genome-wide identification of chemo-sensitive SNP markers in colorectal cancer was conducted by Kim and colleagues [199]. Besides the characterization of the NCI60 panel regarding the mutation status of 24 genes, causally implicated in oncogenesis and drug response [200], the panel was also screened for chemosensitivity associated SNPs. This resulted in several studies dealing with the establishment of pharmacogenetic markers [201, 202].The impact of polymorphisms in genes involved in anticancer drug efficiency, response to chemotherapy and potential side effects has been reviewed elsewhere [203, 204]. Whereas the current knowledge about SNPs provides us with invaluable tools to find and understand significant associations between SNPs and drug response, we do not fully understand the genetic complexity of the attributes, underlying individual variability in drug response. Interpretations of associated studies are complicated by the number of genes, variants in each gene and the frequency of a variant within a population. The location of a variant SNP in the coding region, the regulatory region, or the non-coding region of the genome also affects proteinexpression and function, in a way that is not yet fully understood. Further advances in molecular biology and bioinformatics will make it possible to comprehensively understand the complex influence of SNPs on gene expression, protein expression and finally protein function. This will add its part to the understanding of the complex network of determinants underlying individual response to anticancer therapy.

Furthermore, FISH analysis is routinely performed to assess general cytogenetics and in particular disease-related chromosomal disorders, e.g. chronic myelogenous leukemia, acute lymphoblastic leukemia and Down syndrome. For prediction of response to anticancer therapy, the FISH technique has been primarily used to determine the copy number of the HER-2 gene to select for HER-2 targeted therapies such as trastuzumab and lapatinib in breast cancer. Therefore, the determination of HER-2 gene amplification by FISH technique is widely used in clinical trials evaluating HER-2 targeted therapies [180–183]. In the analysis of response to targeted anticancer therapy, FISH is mainly used to investigate relationships between the copy number of a gene, encoding a target protein and individual response to therapy [184, 185].

### DNA microarray profiling

A DNA microarray is a multiplex technology used to simultaneously measure expression levels of thousands of genes. RT-PCR applications are generally the techniques of choice, based on the enhanced sensitivity with the ability to detect RNA over a seven-log range. This technology has been miniaturized on small silicon chips or glass slides with the feasibility to accommodate over 30,000 oligonucleotides or cDNAs and has thus adapted to high-throughput performance. The huge amount of data produced by those experiments is being analyzed by pattern recognition software, using clustering algorithms for the identification of groups of genes whose expression varies in the same way between groups. Bioinformatic data mining has the potential to reveal unknown patterns of relationships between genes, in context to response mechanisms to anticancer therapy.

The identification of gene sets with a functional role in chemosensitivity may provide assistance in the choice of patient-tailored therapeutic regimens and for therapeutic intervention in drug-resistant disease.

Since more than 100,000 compounds were screened for anticancer activity patterns against the NCI60 cell line panel and the resulting data has revealed information on the mechanisms of action and resistance of those compounds [205–207], several genomic studies have been conducted using the well characterized NCI60 cell line panel as a basis. The p53 tumor suppressor pathway [208], membrane transporters and channels [209], reductase enzyme expression [210], EGFR expression and amplification [211], P450 enzyme expression [212] and MRP expression [213] are some examples for the investigations of relationships between distinct gene expression patterns and response to anticancer therapy based on the NCI60 panel. Among others, Weinstein and colleagues have analyzed gene expression patterns of the NCI60 panel on the basis of activity patterns of compounds [207, 214]. To improve the reliability of gene signatures predictive for chemosensitivity, robust methods for combining microarray expression data with NCI60 chemosensitivity data were developed. Algorithms for predicting chemosensitivity were optimized based on different bioinformatic filter- and cluster approaches [215, 216]. The bioinformatic approach called the co-expression extrapolation (COXEN) algorithm has been shown to be useful in the NCI60 panel to predict chemosensitivity, even in cell lines of histological types not included in panel. That led to the question, whether this approach could be used to predict drug sensitivity in different patient’s primary tumors. A modification of the COXEN algorithm has been demonstrated to be potentially applicable to bypass the intermediate animal model and achieve predictability of response to anticancer therapy in the clinic situation [217].

Results from the NCI60 panel were also observed in other cell line panels, e.g. the classification of drugs based on their modes of action [218]. Based on these results, Nakatsu et al. complemented the JFCR-39 cell line panel and developed an integrated database of chemosensitivity correlated with gene expression for this new cell line panel, called JFCR-45. This revealed candidate genes which may be related to chemosensitivity. To proof this, the ability of these candidate genes to alter chemosensitivity after being individually over-expressed was examined [219]. Another panel consisting of 30 cell lines was also used in a study, which focused on chemoresistance to in vivo concentrations achieved by anticancer drugs. Gene expression patterns provided 76 new candidate genes with associations to multidrug-resistance. This may allow prediction of response to anticancer therapy in a clinical situation [220]. Sekine et al. published in 2007 [221], highlighting genes which potentially regulate chemosensitivity of tumor cell lines to anticancer therapy. Furthermore, comprehensive studies correlating gene expression patterns and chemosensitivity were conducted using human tumor xenografts [88, 89, 222]. A genome-wide study, analyzed gene expression profiles of 85 cancer xenografts in mice that had been established from nine different human organs. The applied cDNA microarray consisted of 23,040 genes, used to study those xenografts. The study resulted in the identification of 1,578 genes whose expression levels correlated significantly with chemosensitivity [223]. All these studies suggest that the combination of unbiased genome-wide chemosensitivity analysis using array-based approaches may identify candidate genes or gene sets with the capacity to predict cancer cell chemosensitivity. It is important, that these candidate genes or gene sets identified in these functional approaches still require extensive validation in vivo before they can be considered as putative biomarkers and find application in the clinical setting. Therefore, several studies were conducted in patient cohorts to identify predictive biomarker for anticancer therapy directly in the clinical setting. Clinical trials were carried out for different tumor entities, e.g. colorectal cancer [224, 225], oesophageal cancer [226], epithelial ovarian cancer [227], pancreatic cancer [228] and breast cancer [229, 230]. These studies reported on gene signatures that may enable prediction of the response to anticancer therapy. Even though these studies were carried out directly in patient cohorts, the resulting predictive signatures have to be validated in independent studies, with a more significant number of patients. The instability of gene expression signatures derived merely from associative studies has been documented [231–233] and contributes to failed attempts to identify gene expression patterns predictive of response to anticancer therapy. Consequently, DNA microarray analyses of small clinical trial cohorts may not yield gene signatures with power sufficient to predict chemosensitivity [234]. Furthermore, tissue sampling and quality have a major impact on profiling results, due to the fact that transcriptional profiles are the sum of mRNA expression contributed by all tissue components [170]. Individual tumor mRNA expression heterogeneity and the varying tumor content in clinical samples, may give a significantly impaired transcriptional profile among samples [235]. The interpretation of microarray results is also difficult in that complex bioanalytic and bioinformatical analysis techniques are used, which are not yet fully standardized. Nonetheless, the analysis of gene expression patterns greatly contributes to the understanding of the complex cellular mechanisms underlying individual response to anticancer therapy.

Currently, commercialized microarray-based multigene assays are already available. For example, the MammaPrint assay (Agendia BV, Amsterdam, The Netherlands) comprising 70 genes, which is currently designed as a pure prognostic assay for women under the age of 61 with either ER-positive or ER-negative, lymph node negative breast cancer [236]. This assay has not yet been shown to be able to predict sensitivity to anticancer treatment. The oncotype DX™ is a 21-gene, prognostic and predictive assay that determines the 10-year risk for disease recurrence in patients with ER-positive, lymph node negative tumors. In contrast, this assay has been reported to predict benefit from tamoxifen treatment in patients with a low or intermediate risk score and benefit from chemotherapy in those with a high-risk score [237]. A combination of several pharmacogenomic gene sets, designed primarily as a predictive test for guiding selection of therapy is called the NuvoSelect™ assay. One of the used gene sets consisting of 30 genes predicts response to preoperative combination treatment with paclitaxel, 5-fluorouracil, doxorubicin, and cyclophosphamide (TFAC). Another gene set predicts clinical outcome after 5 years of endocrine therapy [238].

In summary, the collection of huge databases of gene expression studies will hopefully reveal a comprehensive picture of the genomics of cancer and contribute its part to individualized anticancer therapy.

## Proteomics

**Fig. 5:**
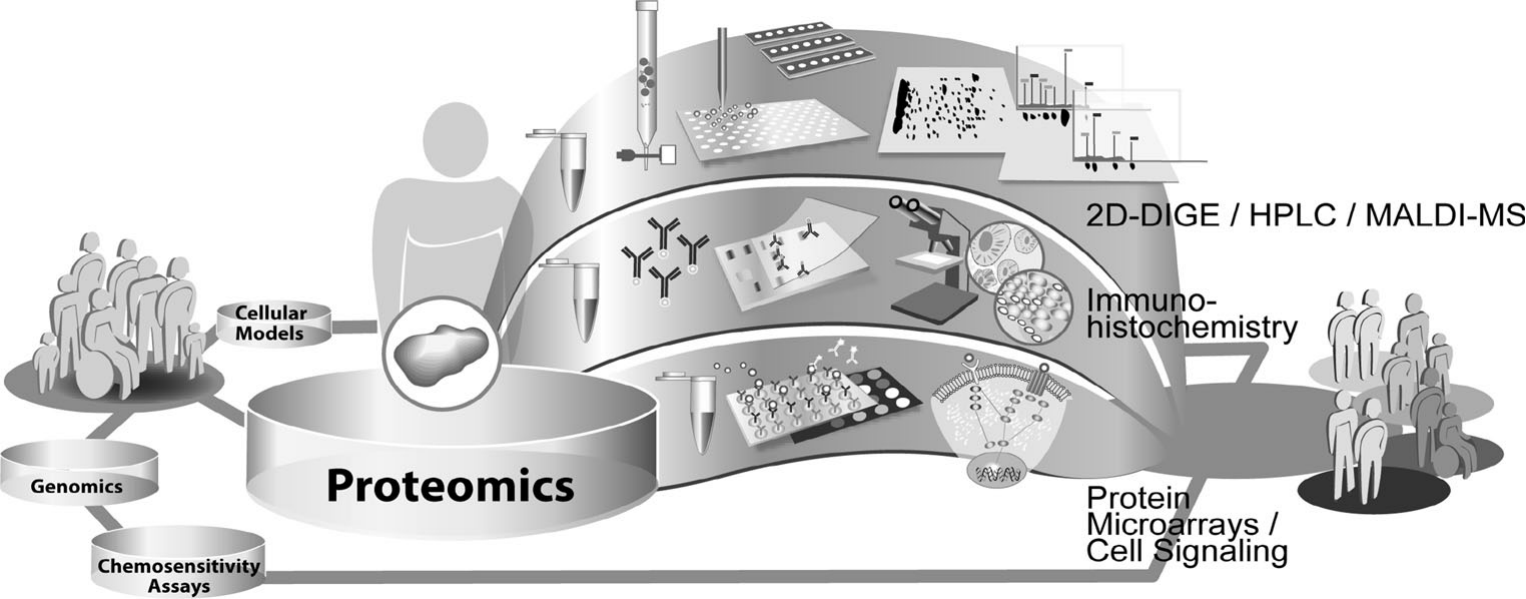
Exemplary illustration of different proteomic approaches used in translational research

Functionally, cancer is a genomic as well as a proteomic disease. While the basic information for the production of proteins is encoded by the genome, only subsets of the possible protein products abundant in the cell are displayed in the genetic code. Finally, the structure of protein products and their functional status often depend on post-translational modifications, such as phosphorylation, glycosylation and proteolytic cleavage that are not reflected in their genomic sequences. Furthermore, gene expression often does not correlate with the protein expression or the functionality of the encoded protein [239, 240]. Since, cellular signal transduction is mostly a post-translationally driven process, it seems obvious to directly investigate the protein-driven signaling cascades by the use of proteomics [241]. Proteomics is a recent member of the ‘omics’ family and describes the study of the wide complement of cellular proteins, their subcellular localization, turnover and interaction with other proteins. In contrast to the genome, the proteome is at a constant flux due to diverse environmental influences. Therefore, the proteome is significantly more challenging to map, compared to the genome [242]. Alterations within the proteome also have a potentially higher functional impact than modifications in the genome, because they are more likely to contribute to a drug-resistant phenotype [243].The analysis of proteins and protein networks in cancer using proteomic technologies is known as oncoproteomics (Fig. 5). Given that the proteome of a cell is responsible for key-biologic processes and therefore also makes up the bulk of pharmaceutical targets, oncoproteomics has the potential to revolutionize clinical practice. This includes cancer diagnosis, development and individualized selection of therapies that target exclusively the cancer-specific protein networks, and real-time assessment of therapeutic efficiency and toxicity. Proteins are traditionally measured using low-throughput techniques such as western blotting, in situ hybridization and immunohistochemical staining [244]. Two-dimensional (2D) gel electrophoresis is a widely used technique in proteomic research, due to its high resolving power that permits simultaneous visualization of primary and post-translationally modified gene products in a single gel [245, 246]. This technology has been used to separate proteins on the basis of their size and charge. In combination with mass spectrometry for protein identification this is a widely used approach for the discovery of several biomarker candidates. Matrix-assisted laser desorption and ionization with time-of-flight detection mass spectrometry (MALDI-TOF), surface-enhanced laser desorption and ionization with time-of-flight spectrometry (SELDI-TOF) and antibody-based protein microarrays are modern methods for a rapid and more sensitive high-throughput detection and identification of both known and unknown proteins [247]. In contrast to mass spectrometry-based biomarker discovery, antibody-based profiling requires prior knowledge of the proteins that are going to be investigated. Therefore, the identification of previously unknown protein biomarker candidates is restricted to MS-based discovery approaches. Antibody-based approaches, such as protein microarrays became more and more important with the introduction of targeted anticancer therapy. These technologies are potentially able to map the activation status of cellular signaling pathways comprehensively, which will have a strong impact on individualized treatment concepts and monitoring of response to therapy. Nearly all proteomic techniques that are usually used for molecular analysis in several biomedical fields are also applied in the study of response to therapy in human cancers [248–250]. Oncoproteomics will play an important role in gaining new insights into cancer development and progression as well as in the discovery and validation of new protein targets for diagnostics and prediction of response to anticancer therapy [251–253]. Furthermore it will be a great challenge to acclimatize proteomic technologies for regular use in clinical laboratories.

### 2D electrophoresis

Two-dimensional gel electrophoresis (2DE) is one of the oldest approaches and one of the most powerful protein separation methods available today. The first-dimensional separation of samples is achieved by isoelectric focusing (IEF), which separates proteins on the basis of their charge. Two types of IEF techniques are currently used: the immobilized pH gradient (IPG) technique; and the non-equilibrium pH gradient gel electrophoresis (NEPHGE). The second-dimensional separation is performed using sodium dodecyl sulfate-polyacrylamide gel electrophoresis [254]. The 2DE provides the capability to qualitatively and quantitatively resolve complex protein mixtures to unique spots [255, 256]. The measured protein patterns can be analyzed using sophisticated, bioinformatical software to reveal those proteins that are differentially expressed between samples.

2D-DIGE is an important proteomic tool, especially for translational research involved in biomarker discovery. When absolute biological variation between samples is the main objective, as it is in biomarker discovery, 2D-DIGE is still one of the methods of choice [257]. Several studies were published, identifying novel prognostic or predictive biomarkers, e.g. biomarkers of drug-resistance [258–261]. First experiments, to study resistance to anticancer therapy using 2DE techniques were performed back in 1986, when Shen et al. [262] investigated the mechanisms of multidrug resistance in human cancer cells. Since then, experimental techniques have continuously been improved and modified for various study designs [263, 264]. For example, Tanaka et al. adapted the 2DE technique for a comparative proteomic analysis of basic proteins. In this study, cancer cell lines were analyzed with regard to their chemosensitivity, using a radical-free and highly reducing method of two-dimensional polyacrylamide gel electrophoresis [265]. This technique is reported to have a superior ability in the separation of basic proteins and the quantification of post-translational modifications, compared to traditional 2DE [266]. Different prefractionation methods, prior to 2DE analysis, as well as various combinations of analysis technique have also been developed to gain detailed knowledge of cellular mechanisms involved in response to anticancer therapy. Based upon these developments, detailed studies of different cellular components and protein signaling networks have also been conducted, e.g. the subcellular proteome [267, 268], the phosphoproteome [269], mitochondrial proteome [270] .Using comparative proteomic approaches, long lists of differentially expressed proteins, potentially involved in chemoresistance mechanisms were published, and reviewed by Zhang et al. [271]. Besides studies based on secondary cell lines, these techniques also found application in the clinical setting [272, 273]. In many studies, biomarker candidates were validated by alternative, more specific techniques such as RT-PCR and Northern blot at the mRNA level or Western blot and immunohistochemistry at the protein level. The identified proteins belonged to a variety of different classes of proteins. However, the limitations of this method include limited reproducibility and inability to detect low abundant proteins [274]. These low levels may result in undetectable proteins which significantly limit the application of this method to clinical samples. The combination of 2DE based with liquid chromatographic (LC) protein separation techniques [e.g. 296] and complete gel-free LC–MS approaches are more and more recognized.

### Chromatographic techniques

An alternative, non-gel-based, protein separation approach to 2DE is Liquid Chromatography (LC) [275]. Basically, the components are separated using two phases, a stationary phase and a mobile phase. The procedure is mainly described by the elution of the different components at different rates, due to a varying affinity to interact with the used matrix, which results in a physicochemical separation. This technology is basically used for protein or peptide separations, prior to MS analysis and has been improved to handle proteomic analyses of complex samples [276]. Various chromatography techniques have been developed as methods for protein separation, e.g. reversed-phase [277], cation exchange [278], anion exchange [279], biphasic ion-exchange [280] or size-exclusion [281]. Single- and multidimensional LC can directly be interfaced with the mass spectrometry (MS), enabling automated analysis of large amounts of data for subsequent protein identification [282]. Another 2D chromatographic strategy termed multidimensional protein identification technology (MudPIT) has been extensively applied to proteomic analysis. Mud-PIT is in principle a technique in which two liquid chromatographic steps are interfaced back-to-back in a fused silica capillary to permit two-dimensional high-performance liquid chromatography, combined with mass spectrometry for protein identification [283, 284]. However, the application of tryptic digestion of proteins in these technologies introduces some limitations. Unfortunately, the tryptic digestion of protein samples results in a loss of basic information about the intact proteins, e.g. post-translational modifications. Furthermore, low abundance proteins from a complex mixture may not be detectable in the presence of various peptides originating from other proteins. Therefore, the separation of intact proteins by liquid chromatography may offer advantages over tryptic approaches and the use of gel-based methods. In general, these technologies show advantages over gel-based techniques with regard to speed, sensitivity, scope of analysis and dynamic range [285]. In the field of oncoproteomics these methods have been integrated in the mass spectrometry-based discovery and characterization of novel biomarker candidates for guiding individualized anticancer therapy (e.g.) [286, 287].

### Mass spectrometry (MALDI TOF MS, SELDI TOF MS)

Mass spectrometry is a method of choice for analytical characterization of potential drug molecules and protein identification. This technology is widely used to detect and identify the chemical composition of samples, after ionization, on the basis of their mass-to-charge ratio (*m*/*z*). As described earlier, mass spectrometry is often combined with different protein separation techniques to discovery of protein biomarker. Many variants of mass spectrometry-based approaches have been developed for gel-free proteomic analysis. These methodologies apply different pre-fractionation techniques, such as selective surface binding (SELDI), magnetic bead pre-fractionation or liquid chromatography (LC-MALDI). The basic principle of the surface-enhanced laser desorption/ionization- time of flight (SELDI-TOF) and the matrix-assisted laser desorption/ionization (MALDI) techniques is the fact that the sample is pulsed with laser energy causing proteins or protein fragments to ionize, and fly through a vacuum tube to the detector plate. Their time of flight is affected by the mass of the particle and its charge (*m*/*z* ratio). The detector plate records the intensity of the signal at a given *m*/*z* value, and a spectrum is generated. The different peaks in the spectrum correspond to different *m*/*z* protein species. SELDI-TOF is a proteomic technology used for the quantitative analysis of protein mixtures after selectively capturing proteins on pretreated surfaces. In contrast to the MALDI technology, the SELDI technology uses selective surfaces for binding a subset of proteins based on absorption, partition, electrostatic interaction or affinity chromatography on a solid-phase protein chip surface. Therefore, stainless steel or aluminum-based chips are coated with chemicals (e.g., anionic, cationic, hydrophobic, hydrophilic, or immobilized metal affinity) or biological substances (e.g., antibodies, antigen binding fragments such as scFv, or receptor) to capture protein samples based on their intrinsic properties. These pre-fractionation steps enable the detection of low abundant proteins. Until now, SELDI has mainly been used to characterize patients at risk of the development of cancer based on the direct analysis of body fluids like serum, plasma, and urine [288–290]. Nonetheless, there are approaches to use SELDI-TOF as a clinical proteomics tool for the identification of protein biomarker candidates, being predictive for response to anticancer therapy [90, 291–293].

In general, MALDI techniques immobilize protein samples in an energy absorbing matrix. The entire repertoire of proteins in the sample interacts with the matrix from which a selected subset of proteins is bound to, a function of the composition of the selected matrix. The matrix chemicals absorb energy, which is subsequently passed to the sample proteins. Protein structural information, such as peptide molecular weight, amino-acid sequence composition, type and location of post-translational modification, could be obtained by MS analysis. Two MS technologies are common and widely used, the matrix-assisted laser desorption ionization time of- flight mass spectrometry (MALDI–TOF–MS) and the electrospray ionization mass spectrometry (ESI–MS). MALDI–TOF–MS generates ions from solid-phase samples and measures their mass in a flight tube, whereas ESI–MS generates ions from liquid samples and measures their mass using either quadrupole or time of flight detector. MALDI–MS is the most commonly used technique for peptide mass fingerprinting [294, 295]. MALDI–MS is a fast, robust, easy to perform, sensitive (low fmol range), and accurate (low ppm range) technology, which can be adapted to high-throughput [296]. LC-MALDI approaches have also been used to identify protein biomarker for the prediction of response to anticancer therapy. These studies were performed using cell lines, as well as patient’s tumor and serum samples [297–301]. Mass spectrometry technologies in combination with protein separation techniques have the ability to investigate complex patterns of protein expression and modification. Despite the complexity of the human proteom, the constantly improved proteomic technologies will ultimately enable the measurement of individual molecular profiles of patients on the protein level, with the potential to guide personalized medicine.

### Immunohistochemistry

Similar to the western blot technology, immunohistochemistry is a well-known method which has developed over the years with respect to reproducibility and sensitivity. In 1941 already, Coons et al. [302] published a paper describing an immunofluorescence technique for detecting cellular antigens in tissue sections, which marked the beginning of immunohistochemistry (IHC). The fundamental concept behind IHC is the detection of antigens within tissue sections using specific antibodies. Once antigen–antibody binding occurs, a colored histochemical reaction becomes visible by light microscopy or in the case of fluorochromes using ultraviolet light. Immunohistochemistry (IHC) has long been used as an adjunctive diagnostic tool in a variety of cancers. It has provided clinicians with correlative insight into potential prognosis and differential diagnosis. The initially simple method of IHC has become more complex over the years. Currently, extremely sensitive methods are available to detect one or multiple antigens simultaneously or even to examine hundreds of tissues in the same section for the presence of a particular Antigen (microarray technology).

Automation using automated slide stainer increased throughput and reproducibility. Automated staining according to very stringent and standardized conditions has become more and more important since the introduction of targeted anticancer therapy, wherein target expression is one of the essential preconditions. For example, HER2 testing has become an important part of the clinical evaluation of all breast cancer patients throughout different countries, and accurate HER2 results are necessary for identifying patients who benefit from HER2-targeted therapy. IHC analysis is deeply integrated in breast cancer treatment by being able to determine the HER-2 status, the testing of progesterone receptor, estrogen receptor and the proliferation marker Ki-67 [303, 304]. Hence IHC is routinely used to predict response to both HER-2 and hormonal targeted therapies, but is not yet suitable for the prediction of either efficiency or toxicity of anticancer drugs.

Furthermore, IHC is often being used to validate findings from alternative proteomic studies. For example the validation of prognostic and predictive protein biomarker candidates derived from cell line experiments is commonly performed in clinical tumor samples [305–307]. The validation of proteomic-based discovery using clinical specimen is reviewed by Hewitt et al. [308]. Although, antibody signals can be directly assigned to cellular localizations and thus laser microdissection is not required, IHC results are nonetheless influenced by pre-analytic tissue processing and antigen retrieval. Inconsistent quality of IHC reagents and antibodies is also discussed to influence robustness of IHC results [309]. Despite automation and knowledge, IHC, still lacks uniformity of technique, appropriate controls, and standardization of antibodies and grading techniques, making it difficult to compare results across institutions, laboratories and experiments. The statistical analysis of IHC-based multiple markers may be complicated by the nonlinear nature of IHC staining, the impact of different slide scoring thresholds for different immunostains and different subcellular localization of markers. Limitations of IHC have been addressed by other techniques, including isotopic labeling and in situ hybridization, which allow for more quantitative analysis of variations in protein expression.

### Protein microarrays

Protein microarrays, one emerging class of proteomic technologies, have broad applications for discovery and quantitative analysis of protein expression patterns [310, 311]. This technology is uniquely suited to generate an overview map of known cellular signaling proteins and their activation status, reflecting the state of information flow through cellular networks in individual specimens. In the simplest sense, protein microarrays are immobilized protein spots [312, 313]. Thus, proteins can be arrayed on solid surfaces, capillary systems or immobilized on beads [314, 315]. The spots may be homogeneous or heterogeneous and may consist of a bait molecule, such as an antibody, a cell or phage lysate, a nucleic acid, drug or a recombinant protein or peptide [316]. In the array, detection is achieved by probing with a tagged antibody, ligand or serum/cell lysate. The most advanced format of this technique is the antibody-microarray, in which the targeted proteins are detected by specific antibodies, which were coated on solid surfaces [317]. The reverse-phase protein microarrays (RPPA) for example, immobilize one sample per array spot, enabling an array to comprise hundreds of different cellular lysates or patient samples. The detection of proteins is conducted using phosphospecific and total protein antibodies to determine the activation status of key signaling molecules. This technology has been widely been used to analyze distinct cellular signaling pathways or to screen cell line panels as well as collections of clinical specimens for disease-related protein expression patterns. For example, Jones et al. [318] comprehensively analyzed the protein interaction network for the ErbB receptor family, which may have implications in epidermal growth factor receptor targeted anticancer therapy. Chan et al. [319], first showed the application of multiplexed reverse-phase protein microarrays to the study of signaling kinetics and pathway delineation in a leukemic T lymphocytes cell line after activation of certain receptors. An example of for the use of RPPA to screen protein expression patterns in cell line panels is a study of Nishizuka et al. [320], screening the NCI60 cell line panel using a reverse-phase protein lysate microarray. A finding from this study was that the patterns of protein expression compared with those obtained for the same genes at the mRNA level showed a striking regularity. Cell-structure-related proteins almost invariably showed a high correlation between mRNA and protein levels across the NCI60 cell lines, whereas non-cell-structure-related proteins showed poor correlations. They also proposed that, this technology can be expected to contribute significantly to the identification of molecular markers and targets for individualized anticancer therapy. On this basis, Ma et al. [321] determined whether proteomic signatures of untreated cancer cells were sufficient for the prediction of drug response using the NCI60 panel. In this study, a machine learning model system was developed to classify cell line chemosensitivity exclusively based on RPPA proteomic profiling. The accuracy of chemosensitivity prediction of all the evaluated 118 anticancer agents was significantly higher (*P* < 0.02) than that of random prediction. This study provided a basis for the prediction of drug response based on protein markers in the untreated tumor. Cell line panels find broad application in the proteomic analysis of individual chemosensitivity and drug discovery [322–325]. Protein microarray platforms that can provide a quantitative, multiplexed read-out for cellular signaling and that can utilize microscopic quantities of tissue specimens for upfront analysis are needed for the implementation of this technology in the clinical situation [326] Therefore, the RPPA format has been improved to be able to measure the abundance of many specific proteins in complex solutions and has been adapted to use of very small amounts of protein, [327]. Thus, this technology is well suited for signal transduction profiling of clinical samples, e.g. biopsy specimens [316, 328, 329]. The identification of critical nodes or interactions within these networks is essential to drug development and the design of individualized anticancer therapy [330], especially with targeted drugs [331, 332]. Using breast cancer as an example, Wulfkuhle et al. [241] stated that, phosphoprotein-driven cellular signaling events represent most of the new molecular targets for anticancer therapy. Therefore, the application of reverse-phase protein microarray technology for the study of ongoing signaling activity within breast tumor specimens holds great potential for elucidating and profiling signaling activity in real-time for patient-tailored therapy. Moreover, their data demonstrate the requirement of laser capture microdissection (LCM) for analysis and reveal the metastasis-specific changes that occur within a new microenvironment. Microdissection should be a necessary component of molecular analysis since dramatic changes within specific protein phosphorylation levels were noted between a majority of the undissected and microdissected samples. Laser capture microdissection technology permits a selection of a homogenous tumor population from a field of normal-appearing cells and vice versa, to improve the accuracy of comparative proteomics studies. Furthermore, Haab et al. [327] noted that, the sensitivity of individual antibody–antigen interactions for any given detection system are highly dependent on the relative abundance of the antigen–antibody species and the binding affinities between the probe antibodies and the immobilized antigens. Liotta et al. [333] reported on the analytical challenges faced by protein arrays and proposed a practical guide for optimizing construction and study design. Additionally, a difficulty is associated with preserving proteins in their biologically active conformation before analysis. This will further limit the application of this technology as a routine proteomic strategy, unless clinical samples are routinely taken by the use of highly specified procedures. The broad application of protein arrays in personalized medicine is also impaired by the costs of producing antibodies and the limited availability of antibodies with high specificity and high affinity for the target. Nevertheless, protein microarrays in combination with technologies such as LCM and high standardization will greatly contribute to the improved description of the multi-factorial network, underlying individual response to anticancer therapy and will allow the design of personalized medicine.

## Discussion

For most of the history of medicine, doctors relied on their senses—mainly vision, hearing, and touch—to diagnose illness and monitor a patient’s condition. Since then, biomedical research has made huge progress in diagnosis and treatment strategies. The traditional trial-and-error practice of medicine is progressively eroding in favor of more precise marker-assisted diagnosis and safer and more effective molecularly guided treatment of disease. The aim of personalized medicine is to tailor disease detection, diagnosis and therapy to each individual´s profile, using molecular profiles to predict disease development, progression, clinical outcome and response to anticancer therapy. Recent advances in high-throughput technologies have raised new opportunities in the fields of personalized- and predictive medicine. Thus, enabling researchers to screen the whole genome, proteome, transcriptome, and metabolome for biomarkers, in tumor tissues and body fluids [334]. In addition, new cellular models such as 3D organoid cultures or spheroid systems opened new opportunities in drug discovery and translational research. These models reflect the in vivo situation much better than common 2D models which are however, well suitable for high-throughput screenings. The introduction of modern technologies such as mass spectrometry and protein and DNA arrays, combined with the understanding of the human genome, has enabled simultaneous examination of thousands of proteins and genes in single experiments. These technologies are capable of performing parallel analysis, in contrast to serial analysis conducted with older methods. Due to the variety of data points, they provide opportunities to identify distinguishing patterns for cancer diagnosis and classification as well as for prediction of response to anticancer therapies. Furthermore, these technologies provide the means by which new tumor markers could be discovered. At the current stage, the molecular prediction of response to anticancer therapy is more exploratory, aiming at advancing scientific knowledge within clinical investigations rather than routine in clinical practice.

Although numerous biomarkers have been discovered, only a handful of them, such as HER2 amplification, BCR-ABL translocation, KRAS, BRAF and EGFR mutations have been validated for the use in the clinical reality [335].

Molecular research in human tumors is currently predominantly performed retrospectively, using residual tissue specimens obtained from surgical resection procedures. Those tissues are used for generating hypotheses regarding the clinical relevance of the observed markers in the studied patient populations, target validation, and assay optimization. Often these tissue samples are obtained by core needle biopsies, e.g. fine needle aspiration, resulting in small sample amounts, which are often insufficient for comprehensive molecular analysis with currently available technologies. Therefore, the miniaturization of new emerging technologies is urgently needed.

Furthermore, several studies have shown that tissue samples change their molecular profiles and start degrading immediately after resection from the patient’s blood supply. Several exogenous factors such as ischemia time, drugs administered during surgery and processing protocols have been identified, which affect the molecular and genetic profiles of human tissue samples before, during and after the surgical resection [336]. We propose that tissue samples that reflect molecular reality are a requirement to enable efficient cancer drug profiling and biomarker discovery [337]. Besides technology-based challenges, regulatory issues are also limiting factors in the development of personalized medicine and predictive biomarkers. The clinical validation of putative functional regulators of drug response will run the risk of failure similar to other biomarker development efforts unless strict reporting guidelines are adhered to. Finally, the NCI-EORTC recommends that predictive biomarker studies require even stricter considerations, requiring validation in large randomized trials with sufficient power to detect drug-specific differences in tumor response [192]. Using, combining and further improving state of the art technologies and establishing stringent guidelines, the individualization of anticancer therapy especially in second-line treatment, will become accomplishable.
